# Intraoperative detection of circulating tumor cells in pulmonary venous blood during metastasectomy for colorectal lung metastases

**DOI:** 10.1038/s41598-018-26410-8

**Published:** 2018-06-08

**Authors:** Uyen-Thao Le, Peter Bronsert, Francesco Picardo, Sabine Riethdorf, Benedikt Haager, Bartosz Rylski, Martin Czerny, Friedhelm Beyersdorf, Sebastian Wiesemann, Klaus Pantel, Bernward Passlick, Jussuf Thomas Kaifi, Severin Schmid

**Affiliations:** 1 Department of Thoracic Surgery, Medical Center – University of Freiburg, Freiburg, Germany; 2 Institute for Surgical Pathology, Medical Center – University of Freiburg, Freiburg, Germany; 3 Laboratory of Molecular Medicine and Biotechnology, Campus Bio-Medico University of Rome, Rome, Italy; 4 Institute for Tumor Biology, University Medical Center Hamburg-Eppendorf, Hamburg, Germany; 5 Department of Cardiovascular Surgery, University Heart Center Freiburg, Freiburg, Germany; 6 Section for Thoracic Surgery, Hugh E. Stephenson Jr., MD, Department of Surgery, Ellis Fischel Cancer Center, University of Missouri, Columbia, USA; 7 Comprehensive Cancer Center Freiburg, Medical Center – University of Freiburg, Freiburg, Germany; 8 Faculty of Medicine, University of Freiburg, Freiburg, Germany

**Keywords:** Prognostic markers, Tumour biomarkers, Surgical oncology

## Abstract

Circulating tumor cells (CTC) have been studied extensively in various tumor types and are a well-established prognosticator in colorectal cancer (CRC). This is the first study to isolate CTC directly from the tumor outflow in secondary lung tumors. For this purpose in 24 patients with CRC who underwent pulmonary metastasectomy in curative intent blood was drawn intraoperatively from the pulmonary vein (tumor outflow). In 22 samples CTC-enumeration was performed using CellSieve-microfilters and immunohistochemical- and Giemsa-staining. Additionally 10 blood samples were analyzed using the CellSearch-System. We could isolate more CTC in pulmonary venous blood (total 41, range 0–15) than in samples taken from the periphery at the same time (total 6, range 0–5, p = 0.09). Tumor positive lymph nodes correlated with presence of CTC in pulmonary venous blood as in all cases CTC were present (p = 0.02). Our findings suggest a tumor cell release from pulmonary metastases in CRC and a correlation of CTC isolated from the tumor outflow with established negative prognostic markers in metastasized CRC. The presented data warrant further investigations regarding the significance of local tumor compartments when analyzing circulating markers and the possibility of tumor cell shedding from secondary lung tumors.

## Introduction

During the course of disease up to one half of patients with colorectal cancer (CRC) develop metastasis with a predilection for the liver and lung^1^. Prognosis in metastasized CRC is often unfavorable, however pulmonary metastasectomy in selected patients is associated with a considerable prognosis with 5-year survival rates between 40 and 70% and sometimes even results in cure^2–4^.

The decision making for treatment modalities is a multidisciplinary challenge and application of local measures to a systemic disease remains controversial and is thought to only be beneficial if there is no dissemination of tumor cells and metastases. Consequently a good selection of patients for surgical therapy is crucial. Current criteria are based solely on clinical observations such as the disease-free interval, tumor burden and general dynamics which are surrogate parameters for a favorable tumor biology^5–7^.

In the recent years different circulating markers have been established such as circulating DNA, circulating RNA and circulating tumor cells (CTC). These biomarkers represent useful tools to characterize metastasized diseases and could aid in selection of suitable candidates for local measures^8–10^. CTCs have been isolated in metastatic as well as localized CRC and have been revealed as strong prognosticators in CRC^11,12^.

Notably, heteroclonality leads to a great variation of molecular pathologic characteristics in pulmonary metastases of CRC and each metastasis might be associated with different features, a varying tumor biology and hence characteristic signature of circulating markers^13,14^. Isolation of these markers directly from the tumor outflow, which in the case of pulmonary metastasectomy is represented by the pulmonary vein, could provide novel insights on differences in the amount and characteristics of CTC in contrast to systemically isolated cells. Furthermore, evidence of tumor cell release by secondary lung tumors which could be capable of seeding new metastases would bolster the argument for local measures in these patients.

This pilot study is the first to investigate CTC from the pulmonary vein in secondary lung tumors, to analyze possible differences in the different compartments regarding CTC count and correlation with clinical and pathological characteristics.

## Results

### Clinical and surgical characteristics

In total, 32 samples from 24 patients with stage IV colorectal carcinoma who underwent pulmonary metastasectomy in curative intent were analyzed in this study. In 22 samples from 17 patients, CTC-enumeration was performed using CellSieve - microfilters and immunohistochemical- and Giemsa-staining. As healthy controls (HC) we included a total of 5 patients in whom samples were taken intraoperatively. Reason for operations in these cases were benign diseases, such as empyema and coronary artery bypass surgery and there were no known preexisting malignant conditions.

Additionally 10 blood samples from a different patient cohort with pulmonary metastasized colorectal cancer were analyzed using the CellSearch-System.

We excluded patient-samples from analysis in which intraoperative blood draw from the pulmonary vein was not possible for surgical reasons.

In 32 operations a total of 80 metastases were resected, a median of 2 (range 1–7) in the CellSieve-cohort and 4 (range 1–8) in the cohort analyzed by the CellSearch-System. The most frequent primary tumor type was rectal cancer which was present in 14 cases (58%).

In 14 (82%) and 7 (100%) cases patients had undergone preoperative chemotherapy in the respective cohorts. Lymph node dissection is standard of care at our center and thus in all metastasectomies which were carried out via thoracotomy lymph node dissection was performed. Median removed lymph nodes or –fragments were 9.5 (range 0–46) and 9 (range 2–51). A total of 17 positive lymph nodes were found in 3 patients in the cohort analyzed by the sized based filter system and no lymph node metastasis were found in the other cohort. Clinical and surgical characteristics are summarized in Table 1.

**Table 1:** Clinical, surgical and follow-up data.

	CellSieve	CellSearch
Patients, N	17	7
Age	65 (43–86)	63 (44–78)
Females	5 (29%)	1 (14%)
Total operations/samples	22	10
Preoperative chemotherapy	14 (82%)	7 (100%)
Metastases resected per operation	2 (1–7)	4 (1–8)
Histology Colon Rectum	8 (47%) 9 (53%)	2 (29%) 5 (71%)
Lymphnodes resected per operation affected/total	9,5 (0–46) 17/272	9 (2–51) 0/139
Follow-up, months	19 (0–24)	4 (1–5)
Tumor-recurrence, N lost to follow-up deceased	10 (59%) 1 (6%) 1 (6%)	1 (14%) 0 0
DFI, months	5 (0–77)	2 (0–24)

Continous data are shown as median with range, count data are presented as frequencies and percentages. *DFI* Disease-free interval.

### CTC-yield in tumor-outflow blood is higher than in systemic blood during pulmonary metastasectomy

**Figure 1:**
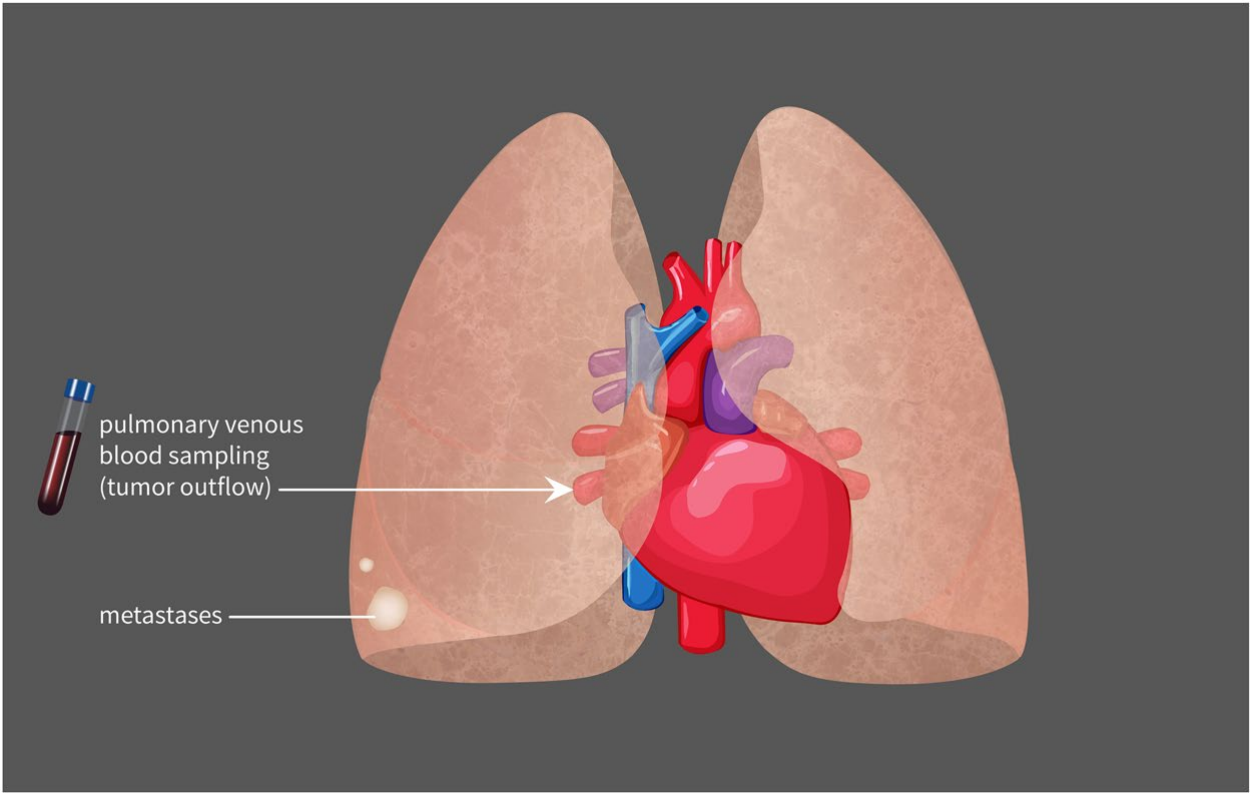
Schematic depiction of the blood sampling from the tumor outflow in secondary lung tumors. Blood samples were drawn from the pulmonary vein of the lobe with the highest tumor burden. ^©^*Nicolas Ritter*.

Multiple previous studies have suggested isolation of CTC in samples drawn from the pulmonary vein to result in higher CTC frequency and count in primary lung tumors compared to samples drawn from the periphery. This is the first study to analysis this matter in secondary tumors in the lung (Fig. 1).

**Figure 2:**
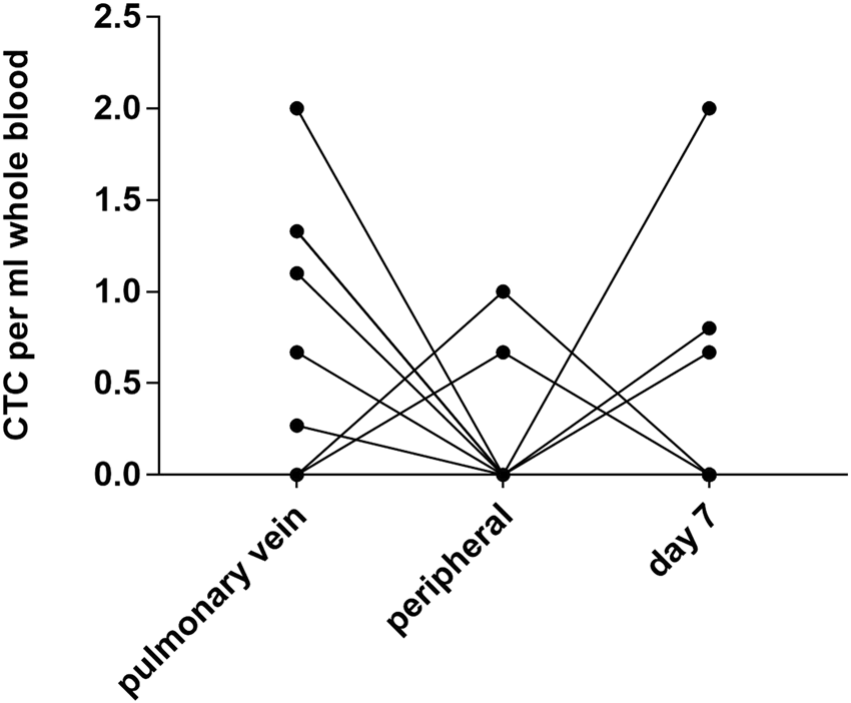
Comparison of CTC numbers per ml whole blood in the different compartments and on day 7 after the operation. More CTC were found in pulmonary venous blood (total 41, range 0–15, mean/ml 0.34) than in samples taken from the periphery at the same time (total 6, range 0–5, mean/ml 0.08, p = 0.09). CTC yield also increased on day 7 after surgery (total 26, range 0–15, mean/ml 0.27, p = 0.41).

In total CTC could be isolated in 9 of 17 patients (53%) whereof 6 of 17 (35%) samples from the pulmonary vein, 2 of 17 (12%) from the periphery at the time of surgery and 3 out of 13 (23%) on day 7 after surgery were positive for CTC (Fig. 2). We could isolate more CTC in pulmonary venous blood (total 41, range 0–15, mean/ml 0.34) than in samples taken from the periphery at the same time (total 6, range 0–5, mean/ml 0.08, p = 0.09). Due to logistic reasons only 12 samples were analyzed on day 7 after operation, nevertheless CTC yield seemed to increase after surgery, however this was not statistically significant (total 26, range 0–15, mean/ml 0.27, p = 0.41).

### Detection of CTC in the tumor-outflow is associated with presence of lymph node metastasis

To evaluate a possible connection of CTC detection and tumor burden, disease dynamics and prognosis we correlated CTC with different clinical and pathologic parameters. Analysis of overall- and disease-free survival did not reveal any differences depending on CTC-positivity or -count. Also there was no correlation of CTC with size or quantity of metastases, previously performed medical treatments as well as disease-free interval or initial tumor stage. Notably, tumor positive thoracic lymph nodes correlated with presence of CTC in pulmonary venous blood as in all cases CTC were present (Fisher’s Exact Test, p = 0.02). Presence of lymph node metastases did not correlate with peripherally isolated CTC.

## Discussion

There is an abundance of retrospective data showing encouraging results after pulmonary and also hepatic metastasectomy in CRC, sometimes even resulting in cure^2,3^. However, there are no prospective data on this matter and whether and how pulmonary metastasectomy could result in a survival benefit remains a matter of debate. As pulmonary metastases and local control, respectively, are seldom responsible for tumor associated death in this patient collective, possible oncological benefits must result from other causes such as cytoreduction and removal of sites which are capable of seeding new metastases.

This is the first study to analyze CTC from the direct tumor outflow in secondary lung tumors in patients undergoing pulmonary metastasectomy. Our aim was to further elucidate disease mechanisms in pulmonary metastasized colorectal cancer, such as tumor cell shedding by metastases and the prognostic value of CTC isolated from the different tumor compartments.

Definition of oligometastasization and thus the indication for local measures is currently solely based on clinical observations and there is a desperate need for robust molecular and or pathologic markers to differentiate varying tumor biologies. CTC have been shown to predict the course of disease in multiple tumor entities as well as for colorectal cancer specifically and could hold potential for adequate individual prognostication as well as for the identification of novel therapeutic molecular targets^11,12,15,16^. The advantage of circulating markers, which in this context often are called “liquid biopsies” is the possibility for sampling from peripheral lines and thus for close and continuous screening. However, previous studies have shown that metastases, even in the same organ and lobes stem from different tumor cell clones and hence show genetic variability. Only in about half of the cases metastases show the same clonal origin as their primary, whereas in the remaining cases they present with a genetically distinct entity^14,17,18^. Analyzing circulating markers solely systemically could possibly not provide the complete information on all the different entities of every single metastasis. Furthermore, selective isolation of CTC could eventually allow backtracking to the origin of the cell, which would especially be of interest in metastasized disease, to evaluate the capability of metastases of seeding secondary tissue.

To provide information on possible differences of circulating markers in the different compartments and to create further insight on the above mentioned issues we evaluated circulating cells from the direct tumor outflow, in this case being the pulmonary vein, in secondary lung tumors. In accordance to previous studies isolating CTC from the tumor outflow in non-small cell lung cancer (NSCLC) we show that CTC yield in is higher when compared to samples which were drawn peripherally^19,20^. The increased yield from the tumor outflow as well as the higher numbers found after resection of the tumor suggests a release of the cells directly from the tumor tissue which could also be amplified by the manipulation during resection. However there is some data suggesting a significance of manipulation for tumor cell dissemination, it remains unclear to what extent surgical techniques impact tumor cell shedding into the circulation and if they do, what the capabilities of the released cells concerning further metastasization are^21^.

The few existing previous studies regarding CTC isolation in the tumor outflow in primary colon cancer and hepatic metastases present similar findings. Tumor outflow of the primary tumor is in this case represented by the mesenteric or portal vein and in case of liver metastases the hepatic vein. Analysis of CTC in the different blood compartments showed a significant higher number in the portohepatic blood circulation compared to peripherally drawn blood samples and CTC numbers dropped after resection of hepatic metastases^22^. Similar observations can be made when analyzing the tumor outflow blood during surgery for primary colorectal cancer^11,22^. Furthermore, a recent trial revealed that CTCs are elevated in the mesenteric vein of primary colorectal cancer, and comparison with peripheral blood demonstrated lower numbers of CTCs, suggesting that the liver captures CTCs before they enter the peripheral circulation^23^. In accordance, liver tumor burden correlates with CTC numbers^24^.

However, these studies were limited to enumeration and there were no further evaluation of the circulating cells which could give insight on their exact origin and significance for metastasization.

In the study at hand we show that presence of CTC in the tumor outflow correlates with presence of thoracic lymph node metastasis, which is one of the best established predictors for short overall- and disease-free survival in patients with metastasized colorectal cancer^2,7,25^. Also, metastasized CRC with thoracic lymph node metastasis is generally considered as disseminated disease and hence respective patients do not qualify for surgical therapy. In common understanding lymph node metastases are not seeded hematogeneously, thus a direct association of these findings is questionable. However, a similar link between lymphonodal metastasization and CTC-detection in the tumor outflow has been shown previously in lung cancers but the underlying mechanisms remain to be determined^26^.

To make reliable statements about CTC origin and significance an isolation technique which allows the researcher further molecular analysis is required. In an attempt for characterization of the isolated cells we analyzed further samples using the CellSearch-system. In our study no CTC could be isolated from peripheral as well as central blood samples using this method. The CellSearch-system is an epithelial cell adhesion molecule (EpCAM)-based enrichment and highly sensitive method for epithelial cells but cannot detect cells that do not express respective markers. Hence cells that underwent epithelial-to-mesenchymal transition or circulating tumor stem cells would not be detected by the system. Furthermore, eligibility for pulmonary metastasectomy predisposes to a relatively benign course of disease with very few metastases mostly confined to one organ and, going in line with most previous studies, CTC-positivity in this patient group using the CellSearch-system is very rare^27^.

### Limitations

Selected patients for pulmonary metastasized colorectal cancer show a relatively benign course of disease and usually have a low metastatatic burden, thus reducing the chance of CTC isolation. To overcome this issue in this pilot study we chose a highly sensitive but not specific method for CTC enumeration in the tumor outflow. Size based filter systems have been shown to increase CTC yield compared to other techniques^27^. Nevertheless, we had a CTC positive sample in the HC group analyzed by the size based filter system. This was a patient who had a high inflammatory burden in pleura and lung due to stage III empyema. As goes in line with previous studies using cytologic criteria alone inflammatory cells can mimic malignant cells and thus lead to false positive results^28^. We have to acknowledge this being a major drawback to these findings, nevertheless deemed this issue acceptable as we are providing first data on this matter. Furthermore, the used methods including CellSearch did not enable us to perform further experiments with the cells which could give insight on their genetic entity and thus origin and possible functions, which limits this study to the observations described above. Finally, recurrence and survival seemed not to be impacted by presence of CTCs in the tumor outflow blood. Yet, overall patient number was low for profound statistical survival analysis and future studies with larger subject volume may potentially show a significant impact on outcome.

## Conclusion

Our findings suggest a tumor cell release from pulmonary metastases in CRC and a correlation of CTC isolated from the tumor outflow with established pathologic markers for negative prognosis and disseminated metastasization. Despite this being a pilot study, our findings stress the importance of the concept of a local tumor compartment in secondary lung tumors when analyzing circulating markers and the possibility of tumor cell shedding from metastases which could lead to further metastasization. The applied methods did not allow us to perform further analysis and thus differentiate the isolated cells, nevertheless the presented findings warrant further investigations regarding this concept.

## Material and Methods

In this prospective observational study we enrolled 24 patients with metastasized colorectal cancer who underwent pulmonary metastasectomy at our clinic as well as 5 healthy controls. The study was approved by our local ethics committee and registered as a clinical trial in the German Registry for Clinical Trials (DRKS-ID: DRKS00007565). All experiments were performed in accordance with relevant guidelines and regulations. All patients signed informed consent before participating in the study.

Inclusion criteria were presence of pulmonary metastases from CRC which were eligible for pulmonary metastasectomy according to common oncologic principles, being absence of extrathoracic metastases, controlled primary (complete resection of the primary tumor and no sign of residual disease) and complete technical resectability of pulmonary metastases (R0) as well as functional eligibility for surgery. Exclusion criteria were the presence of another malignancy and functional or oncologic inoperability.

### Isolation of CTC using CellSieve microfilters

We performed a total of 32 operations of which 30 were carried out via thoracotomy and two using a thoracoscopic approach. Intraoperative sampling was carried out after thoracotomy or trocar placement, respectively, but before metastasectomy. From each patient 7.5 ml of whole blood were drawn from the pulmonary vein from the lobe of the lung with the highest tumor burden. Simultaneously whole blood was drawn from a peripheral line which had been placed preoperatively. Blood was immediately transferred into EDTA-tubes (SARSTEDT AG&Co., Nümbrecht, Germany) to prevent clotting. In healthy controls, blood samples were collected as soon as a pulmonary vein was accessible. Seven days after surgery, again 7.5 ml of patients’ blood was collected into EDTA-containing monovettes via peripheral venous puncture.

For CTC-Isolation each sample was filtered through CellSieve microfilters using the CellSieve CTC Isolation Kit (Creatv MicroTech, Inc., Potomac, MD, USA) and a programmable syringe pump (LEGATO 110; KD Scientific Inc., Holliston, MA, USA) following manufacturer’s instructions. Filters were then covered in 4% paraformaldehyde (Carl Roth GmbH + Co KG, Karlsruhe, Germany) for 10 minutes to fixate the cells and washed in Dulbecco’s phosphate buffered saline (DPBS) (Thermo Fisher Scientific Inc., Waltham, MA, USA).

**Figure 3:**
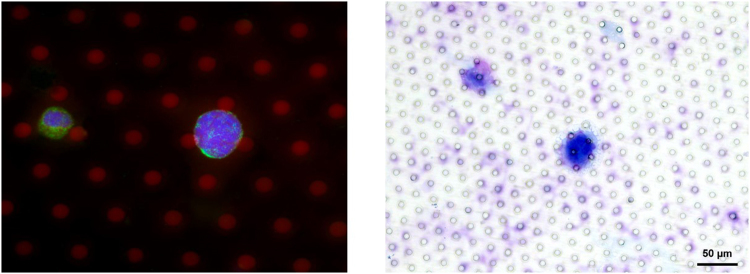
Representative pictures of immunohistochemical (DAPI+, CK8/18/19+, CD45−, left) and Giemsa-staining (right) of CTC isolated from the pulmonary vein in pulmonary metastasized colorectal cancer using a size based filter system.

Immunofluorescence was performed as described before^29^. Stains used were 4′,6-diamidino-2-phenylindole (DAPI) (BIOZOL Diagnostica Vertrieb GmbH, Eching, Germany) for nucleic acid, mouse monoclonal anticytokeratin 8/18/19 (clone 2A4; Abcam plc, Cambridge, UK) and goat antimouse IgG conjugated to DyLight 488 (Thermo Fisher Scientific Inc.) as well as monoclonal mouse antiCD45 conjugated to Alexa Fluor 647 (clone 35-Z6; Santa Cruz Biotechnology, Inc., Dallas, TX, USA). Filters were then put on slides for fluorescence microscopy analysis (BX60; Olympus, Hamburg, Germany) (Fig. 3).

After analysis, filters were removed from the slides by soaking in DPBS. Giemsa-Staining was performed by putting the filters into Hema 3 Solution 1 and 2 for one minute each (Fisher Health Care PROTOCOL Hema 3 Fixative and Solutions; Thermo Fisher Scientific Inc.). Filters were rinsed in PBS and put on slides again. CTC-enumeration was then conducted by an experienced pathologist (PB), who was blinded for the histopathological diagnosis. Cells were considered as CTCs if they (a) an intact cell membrane was detectable, (b) the cells had an enlarged Diameter (larger than 8 µm), (c) formed small or large clusters (not obligate) (d) demonstrated an ill-defined cytoplasm, (e) had irregular, hyperchromatic nuclei, (f) presented a displaced nucleus – plasma relation and (g) prominent nucleioli (not obligate).

### CellSearch-Analysis

From another patient collective 10 samples were analyzed for presence of CTC using the CellSearch system. For this purpose blood samples were drawn as described before, but were transferred into Cellsave Preservative Tubes (Menarini Silicon Biosystems Inc, San Diego, CA, USA). Samples were processed within 96 hours according to the manufacturer’s instructions by a technician trained and certified by the manufacturer to operate the FDA approved CellSearch instrument using the standard CellSearch protocol.

### Statistics

Data were recorded in a database designed in Microsoft Office Excel (Microsoft, Redmond, WA, USA) and GraphPad Prism 7.01 (GraphPad Software Inc., La Jolla, CA, USA) was used for statistical analysis. Categorical and count data are presented as frequencies and percentages. Data sets were tested for normality using the D’Agostino–Pearson omnibus normality Test. In normally distributed data-sets Student’s T-Test, in non-normally distributed data-sets Mann-Whitney-Test was performed. Categorical variables were tested for dependency using Fisher’s Exact Test and the Relative-Risk (RR) as well as the associated confidence intervals were calculated. Results were considered statistically significant if the p value was less than 0.05. A trend was considered when the p value was between 0.05–0.1.

### Data availability

The datasets generated during the current study are available from the corresponding author on reasonable request.
