# Author Correction: Intraoperative detection of circulating tumor cells in pulmonary venous blood during metastasectomy for colorectal lung metastases

**DOI:** 10.1038/s41598-020-64151-9

**Published:** 2020-05-01

**Authors:** Uyen-Thao Le, Peter Bronsert, Francesco Picardo, Sabine Riethdorf, Benedikt Haager, Bartosz Rylski, Martin Czerny, Friedhelm Beyersdorf, Sebastian Wiesemann, Klaus Pantel, Bernward Passlick, Jussuf Thomas Kaifi, Severin Schmid

**Affiliations:** 1 Department of Thoracic Surgery, Medical Center – University of Freiburg, Freiburg, Germany; 2 Institute for Surgical Pathology, Medical Center – University of Freiburg, Freiburg, Germany; 3 Laboratory of Molecular Medicine and Biotechnology, Campus Bio-Medico University of Rome, Rome, Italy; 4 Institute for Tumor Biology, University Medical Center Hamburg-Eppendorf, Hamburg, Germany; 5 Department of Cardiovascular Surgery, University Heart Center Freiburg, Freiburg, Germany; 6 Section for Thoracic Surgery, Hugh E. Stephenson Jr., MD, Department of Surgery, Ellis Fischel Cancer Center, University of Missouri, Columbia, USA; 7 Comprehensive Cancer Center Freiburg, Medical Center – University of Freiburg, Freiburg, Germany; 8 Faculty of Medicine, University of Freiburg, Freiburg, Germany

Correction to: *Scientific Reports* 10.1038/s41598-018-26410-8, published online 08 June 2018

The original version of this Article contained errors. As a mistake, an incorrect subset of data was originally used for the statistical analysis. The table summarising the dataset is now updated, and the results based on this table are corrected throughout the text as follows.

In the Abstract,

“Tumor positive lymph nodes correlated with presence of CTC in pulmonary venous blood as in all cases CTC were present (p  =  0.006).”

now reads:

“Tumor positive lymph nodes correlated with presence of CTC in pulmonary venous blood as in all cases CTC were present (p  =  0.02).”

In Table 1 the ‘Age’, ‘Females’, ‘Preoperative chemotherapy’, ‘Follow-up, months’ and ‘DFI, months’ rows contained incorrect data for either or both of the ‘CellSieve’ and ‘CellSearch’ columns.

In the Results section under the subheading ‘Clinical and surgical characteristics’,

“In 32 operations a total of 80 metastases were resected, a median of 2 (range 1–7) in the CellSieve-cohort and 4 (range 1–8) in the cohort analyzed by the CellSearch-System. The most frequent primary tumor type was rectal cancer which was present in 18 cases (75%).

In 14 (64%) and 10 (100%) cases patients had undergone preoperative chemotherapy in the respective cohorts.”

now reads:

“In 32 operations a total of 80 metastases were resected, a median of 2 (range 1–7) in the CellSieve-cohort and 4 (range 1–8) in the cohort analyzed by the CellSearch-System. The most frequent primary tumor type was rectal cancer which was present in 14 cases (58%).

In 14 (82%) and 7 (100%) cases patients had undergone preoperative chemotherapy in the respective cohorts.”

In addition, in the Results section under the subheading ‘CTC-yield in tumor-outflow blood is higher than in systemic blood during pulmonary metastasectomy’,

“In total CTC could be isolated in 9 of 17 patients (53%) whereof 6 of 17 (35%) samples from the pulmonary vein, 2 of 17 (12%) from the periphery at the time of surgery and 3 out of 12 (25%) on day 7 after surgery were positive for CTC (Fig. 2). We could isolate more CTC in pulmonary venous blood (total 41, range 0–15, mean/ml 0.34) than in samples taken from the periphery at the same time (total 6, range 0–5, mean/ml 0.08, p  =  0.09). Due to logistic reasons only 12 samples were analyzed on day 7 after operation, nevertheless CTC yield seemed to increase after surgery, however this was not statistically significant (total 26, range 0–15, mean/ml 0.20, p  =  0.41).”

now reads:

“In total CTC could be isolated in 9 of 17 patients (53%) whereof 6 of 17 (35%) samples from the pulmonary vein, 2 of 17 (12%) from the periphery at the time of surgery and 3 out of 13 (23%) on day 7 after surgery were positive for CTC (Fig. 2). We could isolate more CTC in pulmonary venous blood (total 41, range 0–15, mean/ml 0.34) than in samples taken from the periphery at the same time (total 6, range 0–5, mean/ml 0.08, p  =  0.09). Due to logistic reasons only 12 samples were analyzed on day 7 after operation, nevertheless CTC yield seemed to increase after surgery, however this was not statistically significant (total 26, range 0–15, mean/ml 0.27, p  =  0.41).”

Furthermore, in the Results section under the subheading ‘Detection of CTC in the tumor-outflow is associated with presence of lymph node metastasis’,

“Notably, tumor positive thoracic lymph nodes correlated with presence of CTC in pulmonary venous blood as in all cases CTC were present (Fisher’s Exact Test, p  =  0.006).”

now reads:

“Notably, tumor positive thoracic lymph nodes correlated with presence of CTC in pulmonary venous blood as in all cases CTC were present (Fisher’s Exact Test, p  =  0.02).”

Finally, in the legend of Figure 2,

“CTC yield also increased on day 7 after surgery (total 26, range 0–15, mean/ml 0.20, p  =  0.41).”

now reads:

“CTC yield also increased on day 7 after surgery (total 26, range 0–15, mean/ml 0.27, p  =  0.41).”

These errors have now been corrected in the PDF and HTML versions of the paper.

